# Current and investigational drugs in early clinical development for portal hypertension

**DOI:** 10.3389/fmed.2022.974182

**Published:** 2022-10-10

**Authors:** Sasan Sakiani, Theo Heller, Christopher Koh

**Affiliations:** ^1^Division of Gastroenterology and Hepatology, Department of Medicine, University of Maryland School of Medicine, Baltimore, Baltimore, MD, United States; ^2^Liver Diseases Branch, Division of Intramural Research, National Institute of Diabetes and Digestive and Kidney Diseases, National Institutes of Health, Bethesda, MD, United States

**Keywords:** portal hypertension, cirrhosis, fibrosis, inflammation, chronic liver disease

## Abstract

**Introduction:**

The development of portal hypertension leads to a majority of complications associated with chronic liver disease. Therefore, adequate treatment of portal hypertension is crucial in the management of such patients. Current treatment options are limited and consist mainly of medications that decrease the hyperdynamic circulation, such as non-selective beta blockers, and treatment of hypervolemia with diuretics. Despite these options, mortality rates have not improved over the last two decades. Newer, more effective treatment options are necessary to help improve survival and quality of life in these patients.

**Areas covered:**

Multiple preclinical models and clinical studies have demonstrated potential efficacy of a variety of new treatment modalities. We introduce treatment options including the use of vasodilation promotors, vasoconstriction inhibitors, anticoagulants, antiangiogenics, and anti-inflammatory drugs. We examine the most recent studies for treatment options within these drug classes and offer insights as to which show the most promise in this field.

**Methodology:**

Published studies that identified novel medical treatment options of portal hypertension were searched using PubMed (https://pubmed.ncbi.nlm.nih.gov/). Clinical trials listed in Clinicaltrials.gov were also searched with a focus on more recent and ongoing studies, including those with completed recruitment. Searching with key terms including “portal hypertension” as well as individually searching specific treatment medications that were listed in other publications was carried out. Finally, current societal guidelines and recent review articles relevant to the management of portal hypertension were evaluated, and listed references of interest were included.

**Conclusion:**

Many ongoing early phase studies demonstrate promising results and may shape the field of portal hypertension management in future. As concrete results become available, larger RCTs will be required before making definitive conclusions regarding safety and efficacy and whether or not they can be incorporated into routine clinical practice. Statins, anticoagulants, and PDE inhibitors have been among the most studied and appear to be most promising.

## Introduction

Portal hypertension (PH) was first described by Leonardo da Vinci in 1511 in his textbook “De humanis corpore” (1), where he wrote “the artery and vein which go from the spleen to the liver become so large, to block the blood coming from the mesenteric vein; the latter vein dilates and becomes tortuous like a snake, that the liver dries and becomes like frozen bran, in color and consistency…” (1). In his description, he incorrectly reported that cirrhosis was due to portal hypertension. The term “portal hypertension” was later introduced by Augustin Gilbert in 1902 while publishing the clinical symptoms of cirrhosis, where he hypothesized that cirrhosis could lead to the development of PH and, in turn, collaterals between the portal and systemic vascular systems (1), and the first portal pressure measurements were made by Thompson in 1937 (1, 2).

Currently, regardless of the etiology of liver disease, it is understood that the development of PH leads to most of the complications associated with end-stage liver disease, and it has even been shown that PH predicts the risk of hepatic decompensation better than liver biopsies in liver transplant recipients (3). Sequelae of portal hypertension include the development of varices and bleeding, ascites, splenomegaly, thrombocytopenia, caput medusae, edema, and hepatic encephalopathy. PH is defined as a hepatic venous pressure gradient (HVPG) of > 5 mmHg, and PH is considered clinically significant when it is ≥ 10 mmHg. Moreover, the HVPG can also predict the risk of further decompensation, with an HVPG ≥ 12 mmHg predictive of increased risk of variceal hemorrhage and ≥ 16 mmHg predictive of increased mortality (4, 5). The HVPG is typically measured by subtracting the free pressure in the hepatic vein (FHVP) from the wedge pressure (WHVP): HVPG = WHVP – FHVP. Less routinely, direct portal pressure measurements can also be performed either *via* interventional radiology or using endoscopic approaches.

When discussing portal hypertension, we generally refer to intrahepatic PH, which is typically due to cirrhosis, but PH can also be seen in non-cirrhotic causes. Pre-hepatic causes such as in portal vein thrombosis, and post-hepatic causes such as right-sided heart failure, Budd–Chiari syndrome, and constrictive pericarditis also lead to increases in portal pressure (Table 1). Understanding the cause of PH is essential as medical management of these conditions generally involves treatment of the underlying etiology. Current medical management for intrahepatic PH generally involves the use of non-selective beta blockers (NSBBs) and somatostatin analogs, which help decrease the hyperdynamic circulation associated with PH through decreased cardiac output and splanchnic vasoconstriction (6, 7). These treatment options do not target the underlying pathophysiology of PH. A meta-analysis performed in 2017 evaluating trends in 30-day and 1-year mortality following hospital admission for hepatic decompensation between 2004 and 2013 demonstrated that while in-hospital mortality has improved, out-of-hospital 30-day survival has not improved with current treatment options and may have even worsened (8), revealing the need for newer, more effective therapies.

**TABLE 1:** Most common causes of portal hypertension.

**Pre-Hepatic**	PV/SV thrombosis Tumor compression of PV/SV Arteriovenous fistulas
**Intra-Hepatic**	
*Pre-sinusoidal*	Schistosomiasis Early PBC Chronic active hepatitis Congenital hepatic fibrosis Sarcoidosis Toxins Portosinusoidal vascular disorders
*Sinusoidal*	Cirrhosis Acute alcoholic hepatitis Cytotoxic drugs Vitamin A hepatotoxicity
*Post-sinusoidal*	Hepatic sinusoidal obstructive syndrome Alcoholic central hyaline sclerosis
**Post-Hepatic**	Right heart failure Pulmonary hypertension Budd-Chiari syndrome Hepatic sinusoidal obstructive syndrome

VOD, veno-occlusive disease; PV, portal vein; SV, splenic vein.

## Pathophysiology of portal hypertension

The portal venous system consists of veins that arise from the gastrointestinal tract, spleen, and pancreas and flow to the liver through the portal vein (PV) (9). The splenic vein and superior mesenteric veins combine to form the main portal vein, which then receives drainage from the left and right gastric veins, as well as the posterior superior pancreaticoduodenal vein. The main portal vein then divides into the left and right portal veins and enters the liver within the porta hepatis. The cystic vein also typically drains directly into the right portal vein (9). The liver receives 25% of the total cardiac output, with the portal vein supplying roughly two-thirds of the blood supply to the liver and the hepatic artery providing the rest (10). In the normal state, sinusoidal pressures remain constant, regardless of changes in blood flow (10).

There are various causes of increased portal pressure, including changes in resistance and blood flow. One of the major consequences of end-stage liver disease is increased portal vascular resistance. This is due to a combination of architectural changes, as well as contraction of vascular smooth muscle cells and activation of stellate cells which when activated promote fibrogenesis (5, 11, 12). Increased blood flow within the portal veins is due to increases in endogenous vasodilators in the splanchnic system (13), which together with the increased resistance in the liver leads to increased pressure within the portal system.

As the pressure in the portal system rises, the body adapts to this increased pressure through the formation of portosystemic collaterals, particularly through the coronary and short gastric veins (14). This, in turn, leads to the formation of varices, including gastroesophageal varices (GEV), which is a result of dilation of pre-existing veins through increased splanchnic nitric oxide production, as well as through neoangiogenesis (14). In addition to splanchnic vasodilation, vasodilation also occurs in the systemic circulation, causing arterial hypotension and activation of the renin–angiotensin–aldosterone system, which can lead to sodium retention, increased blood volume, and increased cardiac output, further leading to exacerbation complications of PH (15).

## Treatment of portal hypertension

### Current treatments

One of the most important interventions in treating PH is treating the underlying etiology of liver disease, such as stopping alcohol use in alcoholic liver disease, treating hepatitis C with direct-acting antivirals in patients with chronic hepatitis C, or weight loss in patients with non-alcoholic steatohepatitis (16–18). Apart from treating the underlying etiology of liver disease, medical management of portal hypertension and its sequelae generally involves controlling the complications once they have already occurred. This includes treatment of portal hypertensive bleeding, including primary and secondary prophylaxis, as well as during acute variceal hemorrhage, in addition to treatment of ascites and edema, hepatic encephalopathy, and hepatorenal syndrome. As noted previously, the current treatment option generally involves the use of vasoconstrictors to ameliorate the hyperdynamic circulation associated with PH. There are few medical options for preventing the progression of portal hypertension.

NSBBs have been used in the management of PH since they were first shown to improve the portal pressure in patients presenting with variceal hemorrhage in 1980 (19). NSBBs, such as nadolol and propranolol, decrease portal pressures by decreasing the cardiac output (*via* β1 receptors) and causing splanchnic vasoconstriction (*via* β2 receptors). Carvedilol is an NSBB which also has anti-α1 adrenergic activity, leading to vasodilation and decreased intrahepatic resistance, and has been shown to improve the HVPG better than propranolol (20). However, due to its ability to cause vasodilation, it is also associated with greater decreases in the mean arterial pressure (MAP), which may limit its use in patients who already have low blood pressures (20). While NSBBs have shown to improve portal pressures and outcomes in patients with cirrhosis and PH, recent studies have emphasized the importance of patient selection and timing of starting these medications. NSBBs have been shown to be more effective in preventing decompensation in patients with clinically significant PH (HVPG ≥ 10 mmHg) than in earlier stages (21). However, given their effect of MAP, NSBBs may lead to lower renal perfusion pressure, leading to acute kidney injury, and it is recommended to avoid their use if the MAP is less than 65 mmHG (22, 23). A retrospective study in 2010 demonstrated increased mortality with NSBBs in patients with decompensated cirrhosis and refractory ascites (24), although a subsequent meta-analysis showed that NSBBs were not associated with a significant increase in mortality in patients with cirrhosis and ascites or refractory ascites (25). In addition, in the PREDESCI (a study on β-blockers to prevent decompensation of cirrhosis with portal hypertension) trial, NSBB use was associated with an overall lower risk of decompensation or death (HR 0.51; 95% CI = 0.26–0.97, *p* = 0.041), mostly through reduction in the incidence of ascites (HR 0.44, 95% CI = 0.20–0.97, *p* = 0.0297) (26). In this study, patients were initially treated with intravenous propranolol with a primary goal of reducing the HVPG by 10%, and those who did not respond were then treated with carvedilol, which led to treatment response in an additional 50% of patients. While it is clear that NSBBs remain the cornerstone of treatment for PH, these medications have their limitations, and additional treatment options are warranted.

There are also several non-medical treatments of PH, including surgical and non-surgical shunts, with the latter being more common. The type of shunts used depends on the anatomy of the patient and goal of therapy, with each having their own benefits and potential complications. A full review of these shunts is beyond the scope of this study, which is geared toward the medical management of PH. Still, it is important to mention these, given the major role they play. Of particular importance, a transjugular intrahepatic portosystemic shunt (TIPS) involves placing a stent connecting the portal vein to the hepatic vein, thereby shunting blood away from the liver and lowering portal pressures. There are many studies showing the benefits of a TIPS in treating PH that is refractory to medical therapy, including in cases of refractory ascites and variceal hemorrhage, and TIPSs have also been shown to improve survival in many settings (27, 28). Potential complications of TIPSs include further hepatic decompensation, worsening hepatic encephalopathy, cardiac failure, TIPS dysfunction, and, in rare cases, death, and thus, proper patient selection is required (28, 29).

### Future targets of treatment

Given what is known about the pathophysiology of PH, novel therapeutic targets can be identified (Table 2). This includes medications that alter intra- and extra-hepatic vasculature and the RAAS, decrease fibrogenesis, decrease ongoing inflammation and cell death, and affect gut microbiota (30). A few treatment options, such as statins and coffee, affect more than one of these targets. More promising potential treatments currently being studied will be reviewed here.

**TABLE 2:** Therapeutic targets under investigation for portal hypertension.

Vasodilation promotors		Examples	Evidence	Warnings
	FXR agonists	OCA GS-9674 PX-20606	Preclinical models only. *need further studies	Increased risk of decompensation in Childs B/C
	Statins	Simvastatin Atorvastatin Fluvastatin	Preclinical models and clinical studies. *ongoing RCTs	Increased risk of myopathy
	PDE inhibitors	Sildenafil Verdenafil Udenafil Tadalafil	Small clinical studies. *need larger clinical studies	
	NO enhancers	AVE 9488 (97) Serelaxin (98) NCX-1000 (99)	Preclinical models only. *ongoing RCT	
**Vasoconstriction inhibitors**				
	ET inhibitors	Ambrisentan (100) Bosentan (101) Macitentan (102) BQ-123 (103)	Small clinical studies. *ongoing clinical trials	
	Urotensin inhibitors	Palusoran (104)	Preclinical models only. *need RCT	
	Eicosanoid inhibitors	Corticosteroids (105) Aspirin (106) Celecoxib (107) Montelukast (108) Ifetroban (109)	Preclinical models only. *ongoing RCT	
	RAA modulators	Captopril (110) Losartan (111) Candesartan (112) Ibesartan (113) Spironolactone (114)	Preclinical models and clinical studies. *possible reduction in PH in compensated cirrhosis *need larger RCTs	
**Anticoagulants**				
	LMWH	Enoxaparin	Preclinical model and small clinical studies. *need larger RCTs	
	Factor Xa inhibitors	Rivaroxaban	Preclinical model only. *ongoing RCT	
**Antiangiogenics**				
	Kinase inhibitors	Sorafenib Regorafenib	Preclinical models and small clinical studies. *need larger RCTs	
**Anti-inflammatory**				
	Caspase inhibitors	Emricasan	Preclinical models and small clinical studies. *need larger RCTs	
	Gut microbiome	Priobiotics Prebiotics Rifaximin Norfloxacin FMT	Small clinical studies. *need larger RCTs	Discrepancy between studies
	Galectin-3 Inhibitors	Belapectin	Small clinical study. *ongoing RCT	
	PPAR agonists	Lanifibranor Pioglitazone Saroglitazar	Small clinical studies. *ongoing RCT	

RCT, randomized control trial; FXR, farnesoid X receptor; PDE, phosphodiesterase; NO, nitric oxide; ET, endothelin; FMT, fecal microbiota transplantation; PPAR, peroxisome proliferator-activated receptor; LMWH, low-molecular weight heparin.

## Medications targeting intra- and extra-hepatic vasculature

Vasoprotective strategies include inhibitors of vasoconstriction, promotors of vasodilation, transcriptional modulators, anticoagulants, and antiangiogenics. Of these, anticoagulants and statins appear to be the most promising treatment options.

### Anticoagulants

As previously noted, the development of PH is partly due to increased intrahepatic resistance to blood flow in addition to splanchnic vasodilation. Intrahepatic resistance is affected by both architectural alterations of the liver parenchyma due to activation of fibrogenic cells, such as hepatic stellate cells and portal myofibroblasts, and changes in the vascular tone (12, 31). In addition, prior studies have demonstrated that there are frequent thrombotic occlusions (microthrombosis) within intrahepatic veins and sinusoids, and that this plays an important part in liver fibrogenesis (32, 33). As such, the role of anticoagulants in the treatment of PH has been evaluated in several studies.

Previously, it was perceived that thrombocytopenia and the elevated prothrombin time associated with cirrhosis meant that these individuals are at increased risk of bleeding from the use of anticoagulants. However, studies have shown that bleeding complications in cirrhosis are more often a direct result of PH, rather than coagulopathy (34). In addition, anticoagulants have been shown to be safe in cirrhotic patients undergoing invasive procedures and have not proven to impact outcomes in gastrointestinal bleeding (35).

Many early studies demonstrated the benefit of heparin in reducing the development of hepatic fibrosis. For example, a study by Li and colleagues demonstrated that long-term administration of low-anticoagulant activity heparin led to a 25% reduction in hepatic fibrosis in rats injected with CCl4 or porcine serum (36). More recently. Cerini et al. demonstrated that the use of enoxaparin in cirrhotic rats led to decreased hepatic fibrin deposition in addition to a reduction in hepatic fibrosis, with a subsequent significant increase in portal flow, reduction in hepatic vascular resistance, and portal pressure (37). Enoxaparin may also have additional benefits, as demonstrated in a randomized controlled study involving 70 patients, randomized to receive enoxaparin for 48 weeks, which revealed significant reduction in portal vein thrombosis and decreased hepatic decompensation (38). However, larger studies should be performed before recommending enoxaparin universally as a treatment option for cirrhosis.

Rivaroxaban, which selectively inhibits factor Xa, is another anticoagulant that has demonstrated promise in the treatment of PH. Unlike enoxaparin, rivaroxaban has the advantage of being orally administered. In 2017, Vilaseca and colleagues demonstrated that rivaroxaban led to reduced hepatic fibrin deposition and decreased the portal pressure in CCL4 cirrhotic rats and in TAA cirrhotic rats (39). Given these promising results, a multicenter prospective randomized trial was started (CIRROXABAN) with the aim of evaluating the effect of rivaroxaban on survival and development of complications of portal hypertension in patients with cirrhosis (clinical trials identifier NCT02643212) (40). The study is still in progress, and thus, further conclusions regarding rivaroxaban cannot be made at this time.

### Statins

Statins consist of a class of drugs primarily aimed at lowering cholesterol through the inhibition of HMG-CoA reductase. However, they have also been shown to have additional benefits which make them an exciting treatment option for patients with chronic liver disease and improving PH (41). This includes improvement in endothelial dysfunction through effects on Kruppel-like factor 2 (KLF-2) and increased nitric oxide (NO) bioavailability through modulation of endothelial NO synthase and enhancing eNOS mRNA stability (41–43). KLF-2 plays a key role in the formation of hepatic fibrosis and endothelial dysfunction as it is endogenously expressed early in the development of cirrhosis in response to vascular dysfunction (44). Simvastatin induces upregulation of KLF-2 expression and improves endothelial dysfunction and prevents ongoing liver damage (44–46). Statins also have anti-inflammatory and anti-oxidant properties and have been shown to decrease hepatic fibrosis in several studies, which can further improve PH (45, 47, 48).

The first study evaluating the effects of statins on PH was published in 2009 by Abraldes et al., an RCT comparing 28 patients receiving simvastatin with 27 patients receiving placebo for 1 month, with a primary endpoint of HVPG reduction of at least 20% (49). In this study, 32% of the simvastatin group reached the primary endpoint compared with 11% (*p* = 0.054) of the placebo group. While there was no significant change in hepatic blood flow, the simvastatin group demonstrated increased indocyanine green clearance, which can be interpreted as improvement in liver perfusion and function.

Since this initial study, there have been multiple other RCTs that have evaluated the effects of statins on PH. A study by Pollo-Flores and colleagues demonstrated a decrease in the HVPG of 2 mmHg compared with 0 mmHg (*p* = 0.02) when comparing simvastatin with placebo (*n* = 14 vs 20, respectively) for 3 months (50). In 2017, a separate study demonstrated that administering simvastatin to patients who did not have adequate reduction in the HVPG with carvedilol alone led to a significant response in an additional 16 of 38 patients (42.1%) (51). In 2018, Bishnu et al. compared 11 patients receiving atorvastatin in addition to NSBBs with 12 patients receiving NSBBs alone and demonstrated there was a reduction in the HVPG by 4.8 mmHg vs 2.6 mmHg (*p* = 0.041), respectively (52).

While these studies have demonstrated measurable improvements in the HVPG with statins, it is unclear whether these changes result in clinically significant outcomes. One of the largest studies was conducted in 2016: a multicenter, placebo-controlled RCT evaluating the effects of statins in patients with cirrhosis (53). In this study, simvastatin use did not result in a significant reduction of rebleeding from esophageal varices compared with placebo (32% vs 39%, *p* = 0.423), although it was associated with a clinically significant improvement in the overall survival (9% vs 22%, *p* = 0.030). Of patient deaths, rebleeding was the cause in 29.4% in the placebo group compared with only 16.7% in the simvastatin group. However, this study also demonstrated an increased incidence of rhabdomyolysis in patients with severe hepatic dysfunction. Another study evaluating statin use in patients with compensated cirrhosis from hepatitis C showed that statin users had lower incidences of decompensation including ascites and variceal hemorrhage and lower mortality than non-users (54).

Based on these studies, while it is difficult to make broad recommendations for routine statin use in all patients with cirrhosis, there does appear to be a portion of patients who do benefit from them. The ongoing SACRED trial (NCT03654053), which is a phase 3, prospective, multicenter, double-blind, randomized clinical trial at 11 Veterans Affairs medical centers, is evaluating the concomitant use of NSBBs along with simvastatin 40 mg/day vs placebo in patients with compensated cirrhosis and clinically significant PH for 24 months, which will hopefully shed more light on this promising treatment option (55).

### Phosphodiesterase-5 (PDE-5) inhibitors

Excessive extra-hepatic nitric oxide (NO) production leads to increased portal blood flow, which aggravates PH. Dysregulation of the NO-cGMP system leading to decreased cGMP can lead to stimulation of stellate cells and myofibroblasts causing progression of fibrosis, as well as contraction of sinusoids, which can worsen PH (56). Therefore, PDE-5 inhibitors may be helpful in treating PH by inhibiting the conversion of cGMP to 5′-GMP, which, in turn, increases cGMP levels and leads to dilation of hepatic sinusoids (57).

Multiple studies have been evaluated the effects of PDE-5 inhibitors on PH. An early study evaluating the effect of vardenafil in cirrhosis by Deibert and colleagues demonstrated increased portal flow and reduction in the HVPG in four of five patients, with a decrease in the portal pressure due to lower sinusoidal resistance (58). Another study by Tandon et al. demonstrated no effect of sildenafil on the HVPG, although it did lower the mean arterial pressure (59). More recently, in an open-label phase 2 study evaluating the effect of udenafil in patients with cirrhosis, a 19.9% reduction in the HVPG (*p* = 0.0006) on day 0 and 15.7% reduction (*p* = 0.040) from day 0 pre-dose to day 6 post-dose were demonstrated, with five of 15 patients having greater than 20% reduction or decrease to < 12 mmHg. In this study, the mean arterial pressure decreased by 6.2 mmHg (*p* = 0.037) in the higher dose (100 mg) group (60). More recently, an open-label study was initiated in 2022 exploring the use of a novel nitric oxide-independent activator of soluble guanylate cyclase in combination with empagliflozin in patients with NASH and compensated cirrhosis with clinical significant portal hypertension (NCT05282121).

Overall, a majority of these studies were able to demonstrate the benefit of PDE-5 inhibitors on PH. More importantly, the improvement in PH tended to be clinically significant, with reductions near 20% in one study. However, this class of medication is also associated with significant decreases in the mean arterial pressure, which can potentially lead to other problems, including worsening ascites and risk of hepatorenal syndrome. Additional studies with large randomized human trials would be needed before making any conclusions regarding PDE-5 inhibitors.

## Medications targeting inflammation

Inflammation associated with chronic liver disease correlates with portal hypertension. This has been shown in acute-on-chronic liver failure, where one study demonstrated a higher HVPG in ACLF and increased severity of intrahepatic resistance correlating with markers of inflammatory response (61), as well as in HIV/HCV, where a reduction in the HVPG by 3.3 ± 2.7 mmHg was seen after HCV treatment with improvement in the HVPG correlating with improvement in inflammatory biomarkers (62).

### Caspases

Caspases are strongly associated with PH through promotion of inflammation, intrahepatic vasoconstriction, splanchnic vasodilation, and apoptosis of hepatocytes (63). Emricasan is a pan-caspase inhibitor that has been demonstrated to decrease caspase-mediated inflammation, fibrosis, liver function, and PH in rat models (64). In a multicenter, open-label study evaluating 23 patients with compensated cirrhosis and PH, emricasan given 25 mg twice daily for 28 days led to a significant decrease in the HVPG in those with severe PH (−3.7 mmHg, *p* = 0.003), with a third of these patients having more than 20% decrease. In addition, aspartate aminotransferase (AST) and alanine aminotransferase (ALT) significantly decreased in all patients (63). More studies will be necessary to determine the clinical usefulness of caspase inhibitors.

### Gut microbiome

Changes in the gut microbiome and bacterial translocation also play an important role in inflammation through augmentation of pro-inflammatory cytokine responses and hence could be another potential therapeutic target (65). However, studies have not demonstrated a clear benefit of modulation of the gut microbiome in order to reduce PH. Rifaximin, a non-absorbable broad-spectrum antibiotic which is routinely prescribed in cirrhosis to treat hepatic encephalopathy, has been described to decrease hepatic decompensation is several small studies (66–68). However, only one RCT directly evaluating the effects of rifaximin on PH in 54 patients failed to demonstrate significant improvement in the HVPG or systemic hemodynamics (69). A small study evaluating the combination of rifaximin and propranolol to propranolol monotherapy demonstrated improved HVPG response rates in the combination group (56.2% vs 87.5%, *p* = 0.034), although only 16 patients were in the combination arm of the study (70). Norfloxacin, another broad-spectrum antibiotic used more commonly in the past, was also evaluated in several studies, but failed to show significant improvement in PH (71).

Apart from antibiotics, other methods of targeting gut microbiome include probiotics, which can potentially restore the bacterial composition in the gut. VSL#3, a probiotic mixture consisting of eight live bacterial strains, has been evaluated in several studies with mixed results. A study evaluating the effect on the HVPG in 17 patients receiving VSL#3 for 6 weeks demonstrated significant reduction of the HVPG, cardiac index, and heart rate, as well as increased systemic vascular resistance (72). However, a second study published around the same time evaluating the HVPG after 2 months of VSL#3 in 17 patients failed to demonstrate any significant change in the HVPG (73). A larger study comparing a total of 94 patients randomized to receiving propranolol plus placebo, norfloxacin, or VSL#3 for 2 months demonstrated significantly increased response rates, defined as reduction of HVPG > 20% from baseline or to < 12 mmHg, in both norfloxacin (−3.4 mmHg) and probiotic (−3.7 mmHg) groups compared with propranolol alone (−2.1 mmHg), with *p* = 0.061. The adjunctive therapy arms were also associated with greater decreases in TNF-alpha, suggesting decreased inflammation (74). Given the small size of these studies and discrepancy in results, additional studies are necessary to further evaluate this treatment option.

Fecal microbiome transplant (FMT) is another potential method of targeting gut microbiome and inflammation. A recent study presented in the 2020 Digital Liver Meeting by Dhiman and colleagues evaluated the effects of FMT in patients with cirrhosis after 180 days. Patients who received FMT had significant improvement in the Child–Turcotte–Pugh score (*p* = 0.01), MELD score (*p* = 0.03), and MELD-Na score (*p* = 0.04), as well as reductions in inflammatory cytokines IL-1 (*p* = 0.01) and IL-6 (*p* = 0.005). However, there was no significant change in 180-day survival (*p* = 0.41) or decompensating events related to PH including control of ascites, new-onset variceal hemorrhage, or breakthrough hepatic encephalopathy (75).

## Other potential therapeutic options

### Kinase inhibitors

JAK2 has been described to be upregulated in patients with cirrhosis, particularly in hepatic stellate cells (76). A recent study by Klein and colleagues demonstrated that mice lacking JAK2 developed less fibrosis and lower portal pressures, suggesting a potential role for JAK2 inhibitors in the treatment of PH (77). Unfortunately, no clinical trials have been published evaluating the use of JAK2 inhibitors, so it is unclear if this would be a feasible option. However, sorafenib, is a multikinase inhibitor commonly used in the treatment of hepatocellular carcinoma, had also been shown to reduce the portal pressure in animal studies (78, 79). In a study by Pinter et al., the use of sorafenib decreased the HVPG 20% below baseline in four of 13 patients (80). Similarly, a study evaluating portal venous flow as measured by MRI in patients receiving sorafenib demonstrated significant reductions of portal venous flow (54% of the mean portal venous flow) in all seven patients receiving sorafenib which returned to baseline after discontinuing sorafenib (81). While this suggests a decrease in the portal pressure, this was not directly measured in this study. Regorafenib, another more potent multikinase inhibitor, has also been evaluated in regard to its effects on PH in animal models and reduced hepatic vascular resistance and PH mainly through its effects on angiogenesis, rather than effects on fibrosis (82). However, hepatotoxic effects during long-term treatment of fibrotic animals was also observed during this study, which may limit its potential use. Overall, further studies are still required before making any conclusions regarding kinase inhibitors.

### FXR agonists

The farnesoid X receptor (FXR) has been shown to play an important role in hepatic inflammation, fibrosis, and vasculature remodeling, and FXR agonists have recently been studied in a variety of diseases, including primary biliary cholangitis and non-alcoholic steatohepatitis (83–86). In addition, animal studies have shown that FXR agonists may also help improve PH. A study evaluating the effects of obeticholic acid, an FXR agonist, on thioacetamide and bile duct-ligated cirrhotic rats demonstrated a significant reduction in PP (87). In a separate animal study, the use of the FXR agonist PX20606 decreased portal pressures in non-cirrhotic partial portal vein-ligated rats and CCL4 cirrhotic rats. This improvement in the portal pressure was due to reduced liver fibrosis, vascular remodeling, and sinusoidal dysfunction. In addition, longer term treatment (14 weeks vs 3 days) was associated with more significant improvements in sinusoidal remodeling and portal pressure (88). Finally, another FXR agonist, cilofexor (GS-9674), was evaluated in a non-alcoholic steatohepatitis rat model and was shown to significantly reduce the portal pressure. The additional of an NSBB, propranolol, further reduced splanchnic inflow, reducing mesenteric hyperperfusion (89).

FXR agonists have already demonstrated benefit in several liver diseases and may potentially play a major role in treating PH if further studies can demonstrate the efficacy and safety in human subjects. Although obeticholic acid (OCA) is approved for the treatment of PBC, it has a black box warning and is associated with decompensation in patients with moderate to severe PBC (90). However, this only relates to OCA, perhaps due to its steroidal structure, and others may not suffer from the same therapeutic issue including non-steroidal FXR agonists that have a different pharmacokinetic profile.

### Galectin-3 inhibitors

Galectin-3 (Gal-3) is a beta-galactoside-binding protein that has been associated with chronic inflammation and fibrogenesis and has been shown to play a role in the severity of NASH (91). In a phase 2b multicenter study evaluating the safety and efficacy of belapectin, a Gal-3 inhibitor, in patients with NASH, cirrhosis, and PH over 52 weeks, there was no significant difference in the HVPG between the belapectin and placebo groups in both 2 mg/kg arm (−0.28 vs 0.10 mmHg, *p* = 1.0) and 8 mg/kg arm (−0.25 vs 0.10 mmHg, *p* = 1.0). However, in a subset of patients who did not have esophageal varices at baseline, 2 mg/kg of belapectin did show a significant reduction in the HVPG (*p* = 0.02), as well as reduced development of new varices (*p* = 0.03). Also, belapectin was well tolerated during this study (92). Given the potential benefit in the subset of patients, a phase 3 study (NAVIGATE) is in process to further evaluate the benefits of belapectin (clinicaltrials.gov identifier: NCT04365868) with a plan to enroll 1,010 participants (93).

### Peroxisome proliferator-activated receptors

Peroxisome proliferator-activated receptors (PPARs) are a group of nuclear transcription factors expressed among various hepatic cell types, including macrophages. PPAR dysregulation in chronic liver disease has been associated with progression of liver disease. In particular, PPAR-γ activation has been shown to promote anti-inflammatory macrophage function (94).

Lanifibranor is a pan-PPAR agonist that has been studied in the treatment of PH and has shown some promise. In the NATIVE study, a phase 2b trial evaluating the effects of lanifibranor in patients with NASH, 48% of patients receiving 1,200 mg and 39% of patients receiving 800 mg had improvement in fibrosis scores (95). A follow-up of the NATIVE study—NATiV3, a phase 3 study—is ongoing with the aim of evaluating the long-term efficacy and safety of lanifibranor in adult patients with NASH and advanced fibrosis (clinicaltrials.gov identifier NCT04849728) (96), although effects on the portal pressure are not a major aim of this study. While additional PPAR agonists such as pioglitazone and saroglitazar have also been studied in the treatment of NASH, none of these have been evaluated in the treatment of PH. Additional studies will be needed to determine if PPAR agonists are a good option for treating PH.

## Conclusion

The development of PH leads to a majority of complications associated with chronic liver disease, including gastric and esophageal varices, ascites, and hepatic encephalopathy. Therefore, adequate treatment of PH is crucial in patients with chronic liver disease. Currently, medical management of PH generally involves dealing with each of these complications as they occur, with few options for preventing the progression of PH. This includes the use of NSBBs and somatostatin analogs, which help decrease the hyperdynamic circulation, as well as diuretics to decrease hypervolemia and antibiotics to lower ammonia-producing gut bacteria.

Given what is known about the pathophysiology of PH, several additional treatment targets have been studied in recent years with promising results. This includes medications that are vasodilators, vasoconstriction inhibitors, anticoagulants, antiangiogenics, and anti-inflammatory drugs. Of these, statins, anticoagulants, and PDE inhibitors have been among the most studied, although they also have significant potential complications, including increased rhabdomyolysis, bleeding complications, and hypotension, respectively. FXR agonists, which have recently been evaluated in the treatment of various chronic liver diseases and have been shown to reduce inflammation and fibrosis, have also shown promise in reducing PH, although are also associated with significant side effects and have a black box warning since they have been shown to increase the risk of decompensation in patients with moderate to severe PBC. Finally, the gut microbiome, which has recently proven to play a major role in many aspects of liver disease, has not yet demonstrated to be an effective target in treating PH with either antibiotics or FMT. Unfortunately, most studies evaluating these and other aforementioned treatment modalities are either small or preclinical models. Larger RCTs are still required to evaluate the safety and efficacy of these medications prior to making any conclusions and incorporating them into routine clinical practice.

In the era of personalized medicine, it is expected that future treatment modalities for the management of portal hypertension will combine current treatment options with one or more newer treatments based on their underlying disease and risk. For example, in a patient with non-alcoholic steatohepatitis, the benefits of starting a statin may greatly outweigh any potential risks, even in advanced stages of cirrhosis. On the other hand, in a patient with alcoholic liver disease and severe hepatic dysfunction, the risk of addition of a statin may be too high risk to have any benefits in PH. Likewise, a patient with cirrhosis and no prior bleeding may benefit from the addition of an anticoagulant, which may help prevent the first variceal bleed, while the risk of starting an anticoagulant may be too high in another patient with significant portal hypertensive gastropathy which may result in bleeding, or in patients with ongoing alcohol use and the tendency to fall.

Over the course of the next 5 years, results of ongoing clinical studies evaluating the safety and efficacy of newer therapies should become available. Guided by these results, clinicians will then be able to tailor available portal hypertension treatment to patients through the addition of new therapeutic options, with or without current treatment strategies, with the ultimate hope of improving survival and quality of life in these patients.

## Author contributions

SS, TH, and CK contributed to concept, design, review of the data, and drafting of the manuscript. All authors approved the final draft of the manuscript.
